# Nonsteroidal Mineralocorticoid Receptor Antagonists in Heart Failure: Mechanistic Basis, Clinical Evidence, and Therapeutic Integration

**DOI:** 10.3390/ddc5020033

**Published:** 2026-05-28

**Authors:** Rami A. Al-Horani, Navneet Goyal

**Affiliations:** Colleges of Pharmacy & Arts and Sciences, Xavier University of Louisiana, New Orleans, LA 70125, USA

**Keywords:** heart failure, HFpEF, HFmrEF, finerenone, nonsteroidal mineralocorticoid receptor antagonists, SGLT2 inhibitors, cardiorenal syndrome

## Abstract

The therapeutic landscape for heart failure (HF), particularly in patients with mildly reduced (HFmrEF) and preserved ejection fraction (HFpEF), has historically been characterized by limited effective disease-modifying options. The recent approval of nonsteroidal mineralocorticoid receptor antagonists (nsMRAs), specifically finerenone, represents a major paradigm shift. This review synthesizes contemporary evidence, including the landmark FINEARTS-HF trial, which demonstrated significant reductions in cardiovascular death and heart failure hospitalizations in patients with left ventricular ejection fraction (LVEF) ≥ 40%. These findings contrast with the neutral overall results and subgroup limitations observed with steroidal MRAs such as spironolactone in the TOPCAT trial. Mechanistic distinctions, cardiorenal benefits, and emerging metabolic effects of finerenone are explored alongside its complementary role with sodium–glucose cotransporter-2 (SGLT2) inhibitors. Practical considerations for implementation, including patient selection, dosing, monitoring, and combination therapy strategies, are discussed. Overall, nsMRAs establish a new foundation for the management of HFmrEF and HFpEF and represent a critical advancement in contemporary heart failure therapeutics.

## 1. Introduction

**Figure 1.**
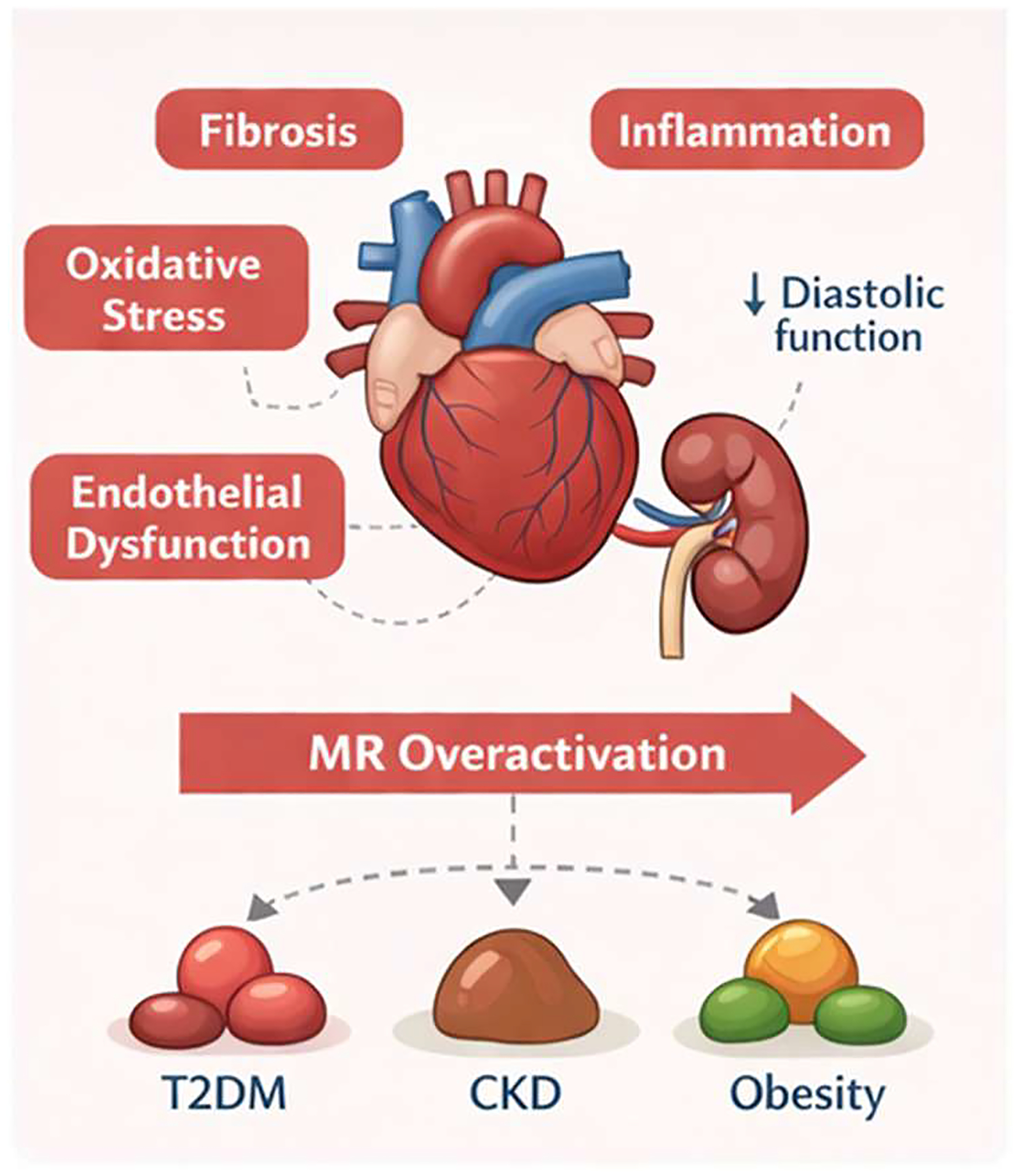
Integrated pathophysiology of heart failure with mildly reduced (HFmrEF) and preserved ejection fraction (HFpEF). The diagram illustrates the interaction between comorbid conditions, systemic inflammation, neurohormonal activation, and cardiac and renal remodeling that drive disease progression. CKD: chronic kidney disease; MR: mineralocorticoid receptor; T2DM: type 2 diabetes mellitus.

Heart failure with preserved (HFpEF; ≥50%) and mildly reduced ejection fraction (HFmrEF; 41–49%) collectively account for more than half of all heart failure cases and are associated with substantial morbidity, mortality, and healthcare utilization [1–3]. These phenotypes are characterized by complex pathophysiology involving diastolic dysfunction, myocardial fibrosis, systemic inflammation, endothelial dysfunction, and a high burden of cardiometabolic comorbidities (Figure 1). Despite their prevalence, therapeutic progress in HFpEF and HFmrEF has historically lagged behind that of heart failure with reduced ejection fraction (HFrEF), where multiple pharmacologic classes have demonstrated clear mortality benefits.

**Figure 2.**
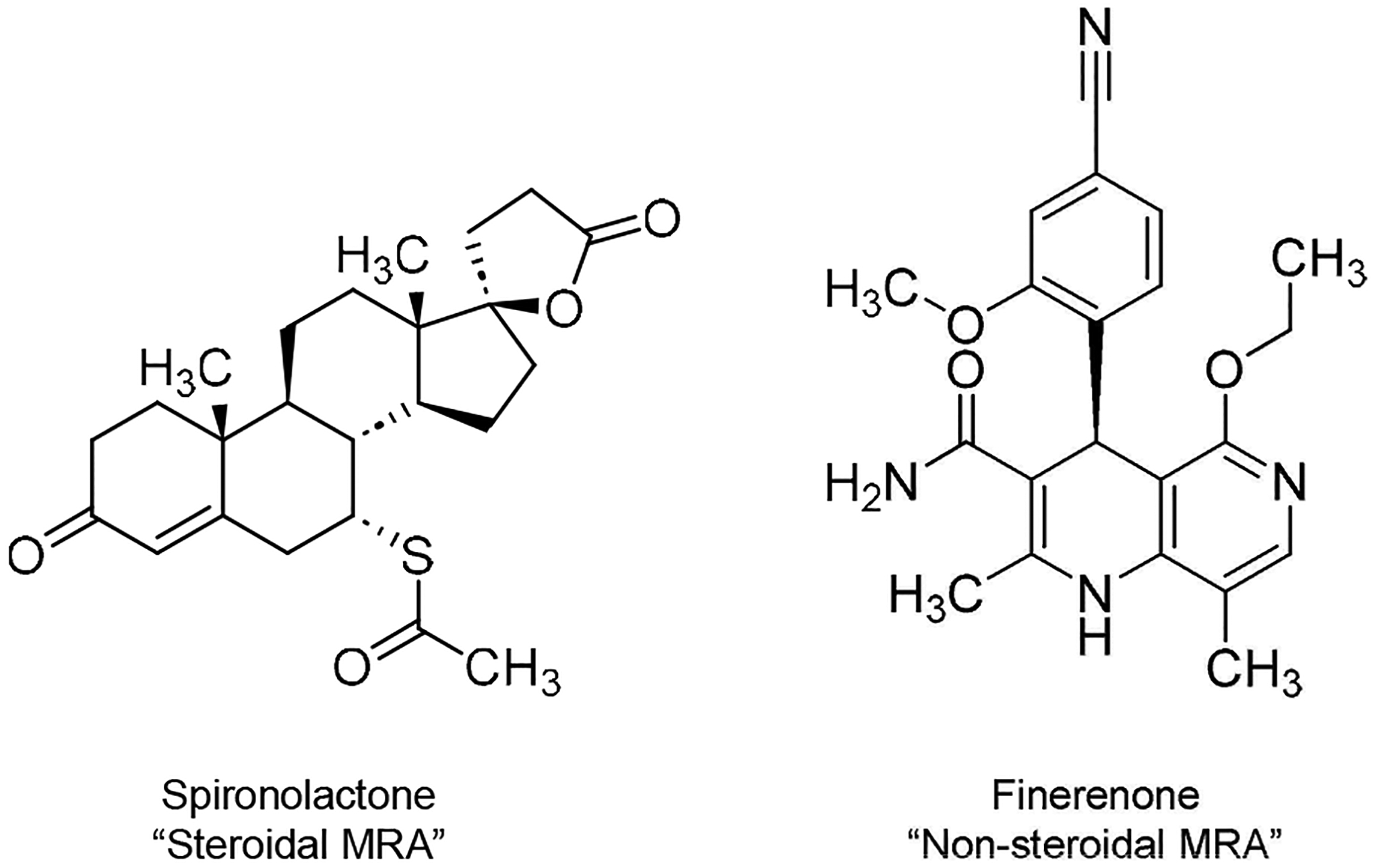
The chemical structures of steroidal and nonsteroidal MRAs.

For many years, management strategies in HFpEF and HFmrEF were largely limited to symptom control with diuretics and optimization of comorbid conditions, with no therapies convincingly shown to reduce major cardiovascular outcomes. The emergence of disease-modifying agents, beginning with SGLT2 inhibitors and now extended by non-steroidal MRAs, marks a fundamental shift in this paradigm. In heart failure, the term “disease-modifying therapy” refers to treatments that alter the natural progression of the disease rather than improve symptoms. These therapies reduce cardiovascular death and heart failure hospitalization, slow cardiac remodeling, preserve renal function, and improve long-term outcomes [1–4]. Disease-modifying therapies are well established for HFrEF but have historically been limited in HFmrEF and HFpEF. The emergence of SGLT2 inhibitors and nonsteroidal MRAs therefore represents a major therapeutic advance in these populations. Finerenone (Figure 2), the first-in-class nsMRA approved for HFmrEF and HFpEF, introduces a targeted approach to mineralocorticoid receptor–mediated inflammation and fibrosis, addressing key drivers of disease progression [4,5].

## 2. Steroidal MRAs and the Limitations of Early Evidence

Mineralocorticoid receptor antagonists have long been a cornerstone of therapy in HFrEF, but their role in HFpEF remained uncertain following the TOPCAT trial [6]. In this large randomized study, spironolactone (Figure 2) failed to significantly reduce the composite endpoint of cardiovascular death, aborted cardiac arrest, or heart failure hospitalization, although a modest reduction in heart failure hospitalizations was observed. The overall hazard ratio of 0.89 (*p* = 0.14) did not reach statistical significance, limiting its clinical adoption in this population. Furthermore, treatment with spironolactone was associated with increased serum creatinine levels and a doubling of the rate of hyperkalemia (18.7%, vs. 9.1 % in the placebo group) [6].

Subsequent analyses revealed important limitations that complicated interpretation. Regional heterogeneity was particularly striking, with patients enrolled in the Americas demonstrating higher event rates and significant benefit from spironolactone, whereas those in Russia and Georgia exhibited unexpectedly low event rates, raising concerns about population differences and trial conduct [7]. Additional post hoc analyses suggested that treatment effects varied according to baseline natriuretic peptide levels, with greater benefit observed in patients with higher levels, supporting the importance of accurate phenotyping [8].

There were no sex differences in outcomes in the control arm or in response to spironolactone for the primary outcome or its components. Spironolactone was associated with decreased all-cause mortality in women (hazard ratio: 0.66; *p* = 0.01) but not in men (*p* interaction = 0.02) [9]. These findings, along with similar attenuation patterns observed with angiotensin receptor blockers and angiotensin receptor–neprilysin inhibitors, underscore the challenge of treating HFpEF and highlight the need for more effective and consistent therapies [10,11].

Reduced therapeutic benefit at higher ejection fractions, particularly above 55–60%, has also been observed with several other neurohormonal therapies in HFpEF. This may reflect the marked heterogeneity of HFpEF. Patients with very high ejection fractions may have less neurohormonal activation and greater contributions from obesity, vascular stiffness, atrial dysfunction, pulmonary hypertension, and systemic inflammation. As a result, therapies targeting neurohormonal pathways may become less effective in these populations. Importantly, finerenone maintained benefit across the full ejection fraction spectrum, including patients with LVEF ≥ 60%, distinguishing it from several earlier therapies [9–11].

## 3. Mechanistic Basis of Nonsteroidal MRAs

The mineralocorticoid receptor plays a central role in cardiovascular and renal pathology, mediating sodium retention, oxidative stress, inflammation, and fibrosis. While steroidal MRAs inhibit this pathway, their clinical utility is limited by off-target effects and pharmacokinetic variability [12–16].

Finerenone is a novel, nonsteroidal MRA specifically designed to overcome the limitations of traditional steroidal MRAs such as spironolactone and eplerenone. It exhibits higher receptor selectivity and affinity, lacks active metabolites, and demonstrates balanced distribution between cardiac and renal tissues, contributing to a more favorable cardiorenal profile. Its lower lipophilicity and higher polarity further influence pharmacokinetics, resulting in improved tissue penetration and reduced off-target accumulation. Comparison is provided in Table 1 [17–28].

At the molecular level, finerenone acts as a potent and selective MR antagonist with inverse agonist activity, suppressing receptor signaling even in the absence of ligand [21,27]. Unlike spironolactone, which behaves as a partial agonist, finerenone reduces recruitment of transcriptional cofactors such as SRC-1, thereby more effectively inhibiting downstream gene transcription [21]. SRC-1 (Steroid Receptor Coactivator-1) is a nuclear transcriptional coactivator that enhances mineralocorticoid receptor–mediated gene expression. Recruitment of SRC-1 promotes transcription of pro-inflammatory and profibrotic genes involved in oxidative stress, myocardial remodeling, collagen deposition, and tubulointerstitial injury. By preventing SRC-1 recruitment and downstream transcriptional activation, finerenone suppresses pathological cardiac and renal remodeling, limits myocardial fibrosis, and reduces inflammatory signaling [21,27].

Structurally classified as a 'bulky-passive' antagonist, finerenone induces conformational changes in the mineralocorticoid receptor that prevent coregulator binding and lead to an unstable receptor–ligand complex [18,21]. Finerenone acts as a “bulky-passive” antagonist because its nonsteroidal structure occupies the ligand-binding domain of the mineralocorticoid receptor and prevents the conformational changes required for coactivator binding and transcriptional activation [17,18]. This stabilizes the receptor in an inactive state and suppresses downstream inflammatory and profibrotic signaling. In contrast, steroidal MRAs such as spironolactone may show partial agonist activity under certain conditions. This distinct mechanism also enables finerenone to inhibit mutant MR variants (e.g., S810L) without paradoxical activation, a limitation observed with steroidal MRAs [18,21].

Preclinical studies demonstrate that finerenone reduces cardiac hypertrophy, natriuretic peptides, and proteinuria more effectively than eplerenone at comparable doses, largely independent of blood pressure lowering [22,28]. Clinically, finerenone has shown comparable efficacy to spironolactone in reducing biomarkers such as BNP and albuminuria, but with a lower incidence of hyperkalemia and renal dysfunction, reflecting its improved safety profile [29–34]. It also produces dose-dependent reductions in albuminuria and modest reductions in nocturnal blood pressure [33,34]. These benefits have been consistently observed in patients with heart failure, chronic kidney disease, and type 2 diabetes, with optimal efficacy at 10–20 mg once daily [29,33,34].

These mechanistic differences translate into enhanced biological effects, including more effective suppression of pro-inflammatory and pro-fibrotic gene expression, which are central drivers of cardiorenal disease progression. Mineralocorticoid receptor overactivation contributes directly to inflammation and fibrosis. MR signaling increases oxidative stress and activates NF-κB and TGF-β pathways [12–16]. This promotes inflammatory cytokine production, fibroblast activation, collagen deposition, and extracellular matrix remodeling [12–18]. These processes contribute to myocardial stiffness, ventricular remodeling, and renal fibrosis. Finerenone suppresses these pathways through potent inverse agonism and inhibition of transcriptional cofactor recruitment. As a result, inflammatory and profibrotic gene expression is reduced. These molecular effects likely contribute to reductions in cardiac hypertrophy, albuminuria, natriuretic peptide levels, and heart failure hospitalizations observed in clinical trials [17,18].

Importantly, these pharmacologic and mechanistic advantages are particularly relevant in conditions such as HFpEF, where diffuse myocardial fibrosis and systemic inflammation play a central role. By more effectively targeting MR-mediated inflammatory and fibrotic pathways while minimizing endocrine side effects (e.g., gynecomastia), finerenone represents a next-generation MRA with a superior benefit–to–risk profile in cardiorenal disease [17,18].

## 4. Evidence from FINEARTS-HF and Pooled Analyses

Evidence from large-scale clinical programs, including FIDELIO-DKD, FIGARO-DKD, and the pivotal FINEARTS-HF trial, has established its efficacy across the cardiorenal spectrum. In pooled analyses encompassing nearly 19,000 participants, including 7008 individuals (36.9%) with mildly reduced or preserved ejection fraction, finerenone reduced the composite of cardiovascular death or heart failure hospitalization (hazard ratio 0.87;95% CI 0.78–0.96; *p* = 0.008), with consistent effects across subgroups defined by kidney function, albuminuria, and glycemic status [35]. This benefit was primarily driven by a reduction in heart failure hospitalizations (hazard ratio 0.84; 95% CI 0.74–0.94; *p* = 0.003), along with a lower incidence of new-onset atrial fibrillation (hazard ratio 0.75; 95% CI 0.58–0.97; *p* = 0.030), although reductions in cardiovascular and all-cause mortality were not statistically significant [35].

**Figure 3.**
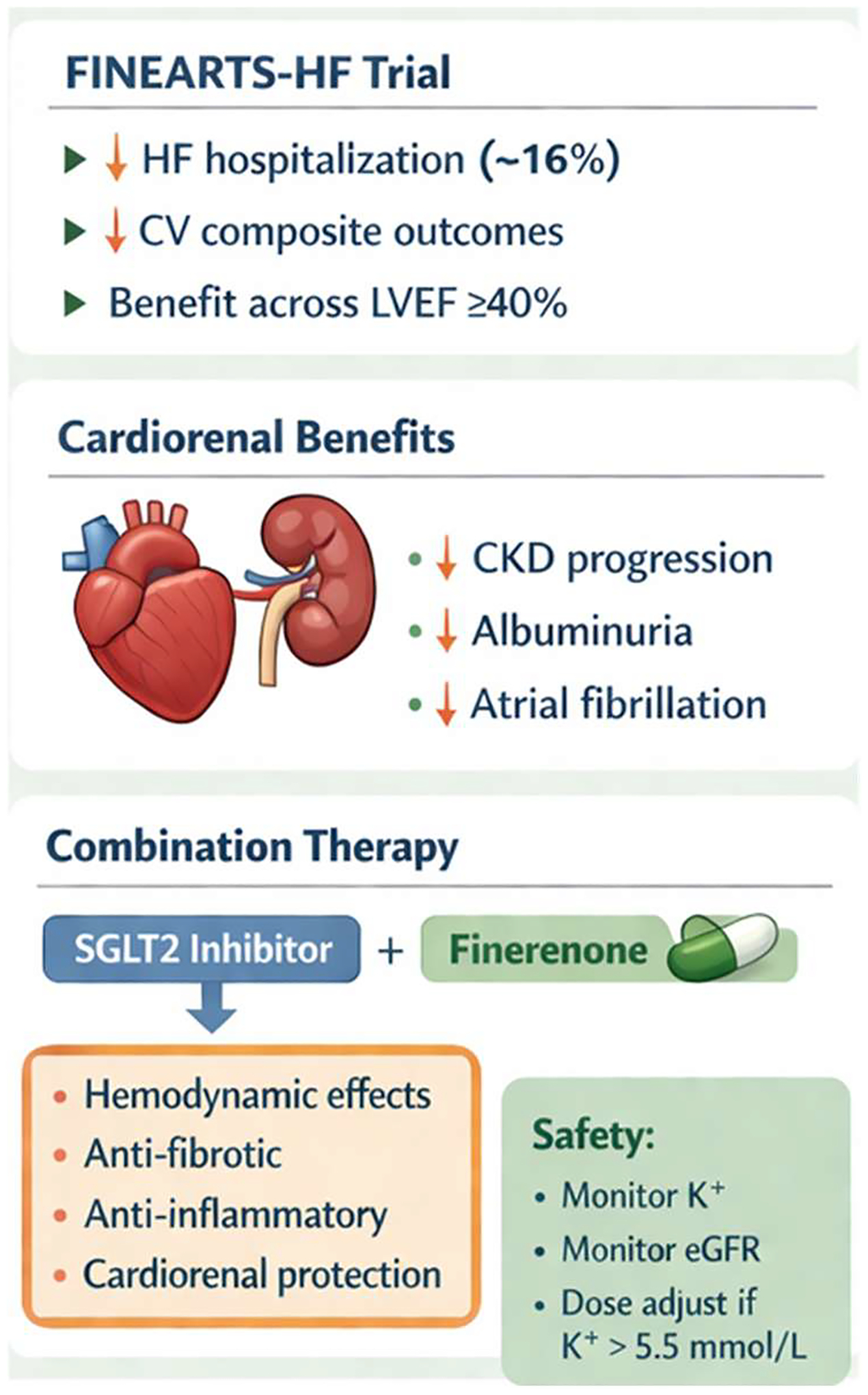
Clinical outcomes and therapeutic integration of finerenone in heart failure with mildly reduced and preserved ejection fraction. The figure summarizes the effects of finerenone on cardiovascular outcomes, renal protection, inflammation, fibrosis, and combination therapy with SGLT2 inhibitors. CKD: chronic kidney disease; CV: cardiovascular; eGFR: estimated glomerular filtration rate; HF: heart failure; LVEF: left ventricular ejection fraction; SGLT2: sodium–glucose cotransporter-2.

Specifically, the FINEARTS-HF trial represents a landmark phase 3 evaluation in this population, enrolling 6001 patients with symptomatic heart failure, left ventricular ejection fraction ≥ 40%, structural heart disease, and elevated natriuretic peptides, including both ambulatory patients and those recently hospitalized, thereby reflecting real-world clinical populations [29]. Over a median follow-up of 32 months, finerenone significantly reduced the primary composite endpoint of total worsening heart failure events and cardiovascular death (rate ratio 0.84; 95% CI 0.74–0.95; *p* = 0.007), corresponding to an approximate 16% relative risk reduction (Figure 3) [29]. The total number of worsening heart failure events was reduced from 1024 in the placebo group to 842 in the finerenone group (rate ratio 0.82; 95% CI 0.71–0.94; *p* = 0.006) [29]. Cardiovascular death occurred in 8.1% of patients receiving finerenone compared with 8.7% in the placebo group (hazard ratio 0.93; 95% CI 0.78–1.11) [29]. Importantly, the therapeutic benefit was consistent across the full ejection fraction spectrum, including patients with values ≥ 60%, distinguishing finerenone from prior therapies that demonstrated attenuated efficacy at higher ejection fractions. Subsequent analyses further suggested meaningful extensions in event-free survival, reinforcing the clinical relevance of these findings [30].

From a safety perspective, finerenone was generally well tolerated but associated with predictable changes in potassium homeostasis. Serum potassium increased modestly, with a median difference of +0.19 mmol/L at 1 month and +0.23 mmol/L at 3 months compared with placebo, persisting throughout follow-up [31]. The risk of hyperkalemia (serum potassium > 5.5 mmol/L) was increased (hazard ratio 2.16; 95% CI 1.83–2.56; *p* < 0.001), whereas the risk of hypokalemia (<3.5 mmol/L) was significantly reduced (hazard ratio 0.46; 95% CI 0.38–0.56; *p* < 0.001) [31]. Notably, both low and high potassium levels were independently associated with worse clinical outcomes; however, the overall treatment benefit was preserved even among patients who developed hyperkalemia, provided that appropriate monitoring and dose adjustments were implemented [31]. Importantly, there was no increase in hyperkalemia-related hospitalization or mortality [29].

Beyond heart failure, finerenone has demonstrated substantial cardiorenal benefits in patients with type 2 diabetes and chronic kidney disease. In FIDELIO-DKD and FIGARO-DKD, finerenone significantly reduced the risk of kidney disease progression and cardiovascular events, findings that were confirmed in the pooled FIDELITY analysis [17,32–34]. Emerging evidence also suggests potential metabolic effects, including a reduced incidence of new-onset diabetes in patients without baseline disease, further supporting a broader role for mineralocorticoid receptor antagonism in cardiometabolic regulation [34,36,37].

Overall, these data demonstrate that finerenone provides a consistent and clinically meaningful reduction in heart failure events, particularly hospitalizations, across a broad population with mildly reduced or preserved ejection fraction, with a manageable safety profile when potassium is appropriately monitored, thereby reinforcing its role within the evolving cardiorenal–metabolic therapeutic paradigm.

## 5. Integration with SGLT2 Inhibitors

SGLT2 inhibitors have emerged as foundational therapy in HFmrEF and HFpEF following the EMPEROR-Preserved and DELIVER trials [38,39]. Finerenone provides complementary mechanisms of action, targeting fibrosis and inflammation, and can be effectively combined with SGLT2 inhibitors (Figure 3). Clinical evidence demonstrates that the efficacy of finerenone is independent of baseline SGLT2 inhibitor use, with additive benefits observed in combination therapy [40,41]. Importantly, while the combination may modestly increase serum potassium levels, it appears to reduce the incidence of severe hyperkalemia, likely due to improved renal handling of potassium. Ongoing trials such as CONFIRMATION are evaluating early combination strategies in high-risk populations, aiming to define optimal sequencing and timing of therapy [42].

Finerenone and SGLT2 inhibitors target complementary pathways within the cardiorenal-metabolic axis. SGLT2 inhibitors primarily improve hemodynamic and metabolic function through natriuresis, osmotic diuresis, reduced intraglomerular pressure, and improved myocardial energetics [38,39]. In contrast, finerenone directly suppresses mineralocorticoid receptor-mediated inflammation, oxidative stress, fibroblast activation, and fibrosis [17,18]. Combination therapy may therefore simultaneously reduce hemodynamic stress, renal injury, metabolic dysfunction, and structural remodeling. Emerging evidence also suggests that SGLT2 inhibitors may reduce severe hyperkalemia risk by improving renal potassium handling [42].

Glucagon-like peptide-1 receptor agonists (GLP-1 RAs) are also emerging as important therapies in patients with obesity, type 2 diabetes mellitus, chronic kidney disease, and HFpEF. These agents improve glycemic control, promote weight loss, and reduce systemic inflammation. Recent evidence suggests that GLP-1 RAs and finerenone may provide complementary cardiovascular and renal protection through overlapping anti-inflammatory and metabolic effects. A recent systematic review and meta-analysis reported favorable cardiovascular and renal outcomes with these therapies in type 2 diabetes mellitus. These findings support a multidrug cardiorenal-metabolic treatment strategy, although dedicated combination trials are still needed [43].

## 6. Clinical Implementation

The integration of finerenone into clinical practice requires a structured approach to patient selection and monitoring. Eligible patients include those with symptomatic HFmrEF or HFpEF, elevated natriuretic peptides, and evidence of structural heart disease. Patients with diabetes, chronic kidney disease, or recent hospitalization may derive particular benefit.

Baseline assessment should include renal function and serum potassium. Dosing is guided by eGFR, with lower starting doses in patients with moderate renal impairment. Monitoring should be performed at one month after initiation and periodically thereafter, with more frequent assessments in high-risk patients. The post-discharge period following hospitalization represents a critical opportunity to initiate therapy and close gaps in guideline-directed medical treatment.

Hyperkalemia remains the primary safety concern with MRAs; however, finerenone demonstrates a favorable safety profile when used with appropriate monitoring. Dose adjustment is recommended for potassium levels exceeding 5.5 mmol/L, and discontinuation is reserved for more severe elevations. A transient decline in eGFR is commonly observed following initiation but should not prompt discontinuation, as long-term data demonstrate renal protection. Clinicians are cautioned against excessive conservatism and premature discontinuation of effective therapies, as this may adversely impact outcomes. Finerenone is well tolerated across age groups, including elderly patients, and does not exhibit the endocrine side effects associated with steroidal MRAs.

Finerenone dosing should be individualized according to kidney function and serum potassium levels. Prior to initiation, serum potassium should generally be ≤5.0 mmol/L. In patients with chronic kidney disease associated with type 2 diabetes, clinical trials excluded individuals with baseline serum potassium > 4.8 mmol/L; however, initiation in patients with potassium levels between 4.8 and 5.0 mmol/L may be considered with close monitoring during the first month of therapy. Finerenone is typically initiated at 20 mg once daily in patients with an eGFR ≥ 60 mL/min/1.73 m^2^ and at 10 mg once daily in those with an eGFR between 25 and 59 mL/min/1.73 m^2^. Use is generally not recommended in patients with an eGFR < 25 mL/min/1.73 m^2^. Maintenance dosing should be guided by repeat assessment of serum potassium and renal function approximately 4 weeks after initiation or dose adjustment, with periodic monitoring thereafter. If serum potassium remains ≤ 4.8 mmol/L, dose escalation to 20 mg once daily may be considered. If serum potassium rises above 5.5 mmol/L, finerenone should be temporarily withheld. Therapy may be restarted at 10 mg once daily once serum potassium decreases to ≤5.0 mmol/L. In addition, if eGFR declines by >30% compared with the previous measurement, maintenance of the 10 mg dose is recommended rather than dose escalation. Careful monitoring is particularly important in elderly patients and in those receiving concomitant therapies that may increase potassium levels [17,33,44].

Given its mechanism, the drug reduces sodium retention, inflammation, and fibrosis in both renal and cardiovascular tissues. Pharmacokinetically, it exhibits moderate bioavailability (~44%), high protein binding (~92%), a short half-life (2–3 h), and metabolism primarily via CYP3A4. Finerenone is contraindicated with strong CYP3A4 inhibitors and in patients with adrenal insufficiency, and caution is required with drugs that increase potassium levels. Routine monitoring of serum potassium and renal function at baseline, after initiation or dose adjustment, and periodically thereafter is essential to ensure safe use [45].

## 7. Conclusions and Future Directions

The emergence of nonsteroidal MRAs, particularly finerenone, represents a transformative advance in the management of heart failure with mildly reduced and preserved ejection fraction. For the first time, robust, phase 3 clinical trial evidence has demonstrated that mineralocorticoid receptor antagonism can significantly reduce major cardiovascular outcomes across the full spectrum of ejection fraction ≥ 40%. This contrasts with the inconsistent and subgroup-dependent benefits observed with steroidal MRAs and establishes finerenone as a disease-modifying therapy in a population historically lacking effective options.

The clinical impact of finerenone extends beyond heart failure alone. Its demonstrated benefits in chronic kidney disease and type 2 diabetes, coupled with emerging evidence of metabolic effects such as reduced incidence of new-onset diabetes, position it as a central component of an integrated cardiorenal-metabolic therapeutic strategy. When combined with SGLT2 inhibitors, finerenone offers complementary and potentially synergistic mechanisms, targeting inflammation, fibrosis, and hemodynamic stress while maintaining an acceptable safety profile. This combination represents a foundational approach to contemporary guideline-directed medical therapy in HFmrEF and HFpEF.

Successful implementation of nonsteroidal MRAs in clinical practice requires careful patient selection, appropriate dosing guided by renal function, and vigilant monitoring of serum potassium and kidney function. Importantly, clinicians should avoid premature discontinuation in response to modest, expected changes in eGFR or potassium, as long-term data support sustained cardiorenal benefits. The post-hospitalization period represents a particularly important opportunity to initiate therapy and close gaps in evidence-based treatment.

Looking forward, ongoing and future investigations will further refine the role of finerenone within heart failure management. Trials evaluating early combination strategies with SGLT2 inhibitors, such as CONFIRMATION, are expected to clarify optimal sequencing and timing of therapy. Additional research is needed to identify biomarkers predictive of response, better characterize benefits in emerging phenotypes such as heart failure with improved ejection fraction, and explore the full extent of metabolic and anti-inflammatory effects.

In summary, nonsteroidal MRAs have redefined the therapeutic landscape of HFmrEF and HFpEF. As evidence continues to evolve, finerenone is poised to become a cornerstone of therapy, offering meaningful improvements in clinical outcomes for a broad and growing population of patients with heart failure.

## Figures and Tables

**Table 1.** Key pharmacologic and mechanistic differences between steroidal MRAs and the nonsteroidal MRA finerenone.

Feature	Steroidal MRAs (Spironolactone/Eplerenone)	Finerenone (Nonsteroidal MRA)
**Generation and selectivity**	Spironolactone is a 1st-generation MRA with potent but relatively nonselective MR antagonism. Eplerenone is a 2nd-generation agent with improved selectivity but lower potency.	Finerenone is a 3rd-generation nonsteroidal MRA with high potency and marked receptor selectivity [17–20].
**Mode of action**	Steroidal MRAs primarily act as passive antagonists. Partial agonistic activity may occur under certain conditions.	Finerenone acts as a potent “bulky-passive” antagonist and inverse agonist, stabilizing the MR in an inactive conformation [21,27].
**Tissue distribution**	Spironolactone and eplerenone demonstrate greater accumulation in renal tissue than in cardiac tissue.	Finerenone shows a more balanced distribution between the kidney and heart, supporting broader cardiorenal activity [23,25].
**Pharmacokinetics**	Spironolactone generates multiple active metabolites with prolonged half-lives. Eplerenone has no active metabolites and a half-life of approximately 4–6 h.	Finerenone has no active metabolites and exhibits a relatively short half-life, allowing more predictable pharmacokinetics and reduced tissue accumulation [25,26].
**Cofactor recruitment in the absence of aldosterone**	Spironolactone and eplerenone may demonstrate partial agonistic cofactor recruitment in vitro.	Finerenone acts as an inverse agonist and suppresses cofactor recruitment even in the absence of aldosterone [21].
**Cofactor recruitment in the presence of aldosterone**	Steroidal MRAs inhibit cofactor recruitment but with less potency.	Finerenone more effectively blocks MR cofactor binding and promotes corepressor recruitment, resulting in stronger transcriptional suppression [21,27].
**Activity at mutated MR variants (e.g., S810L)**	Spironolactone and eplerenone may paradoxically activate mutated MR variants in vitro.	Finerenone maintains antagonist activity without paradoxical activation of mutant receptors [18,21].
**Effects on cardiac inflammation and fibrosis**	Eplerenone reduces inflammation and fibrosis, although effects may be less pronounced at equinatriuretic doses.	Finerenone demonstrates stronger inhibition of inflammatory and fibrotic pathways in experimental cardiac fibrosis models [22,28].
**Effects on renal inflammation and fibrosis**	Eplerenone reduces blood pressure and provides partial renal protection, but with less reduction in proteinuria and structural renal injury.	Finerenone provides greater protection against renal and cardiac injury, stronger suppression of pro-inflammatory and profibrotic markers, and more effective reduction in structural remodeling [22,28].
**Blood pressure effects**	Steroidal MRAs produce clinically significant antihypertensive effects.	Finerenone exerts more modest blood pressure reduction, suggesting benefits beyond hemodynamic mechanisms alone [22,28].
**Clinical safety profile**	Steroidal MRAs are associated with higher rates of endocrine adverse effects and hyperkalemia.	Finerenone demonstrates lower risk of endocrine adverse effects, including gynecomastia, with improved cardiorenal tolerability [17,28].

## Data Availability

No new data were created or analyzed in this study.

## Abbreviations

The following abbreviations are used in this manuscript:

BNP: B-type natriuretic peptide
BP: Blood pressure
CKD: Chronic Kidney Disease
CYP3A4: Cytochrome P450 3A4
eGFR: Estimated glomerular filtration rate
GLP-1 Ras: Glucagon-like peptide-1 receptor agonists
HF: Heart failure
HFpEF: Heart failure with preserved ejection fraction
HFmrEF: Heart failure with mildly reduced ejection fraction
HFrEF: Heart failure with reduced ejection fraction
LVEF: Left ventricular ejection fraction
MRA: Mineralocorticoid receptor antagonist
nsMRA: Nonsteroidal mineralocorticoid receptor antagonist
SGLT2: Sodium–glucose cotransporter-2
